# Epoxyalcohol Synthase Branch of Lipoxygenase Cascade

**DOI:** 10.3390/cimb46010053

**Published:** 2024-01-18

**Authors:** Yana Y. Toporkova, Elena O. Smirnova, Svetlana S. Gorina

**Affiliations:** Kazan Institute of Biochemistry and Biophysics, FRC Kazan Scientific Center of RAS, P.O. Box 261, 420111 Kazan, Russia; ye.o.smirnova@gmail.com (E.O.S.); gsvetlana87@gmail.com (S.S.G.)

**Keywords:** fatty acid oxidation, lipoxygenase cascade, cytochromes P450, CYP74 clan, epoxyalcohol synthase, allene oxide synthase, hydroperoxide lyase, divinyl ether synthase, epoxyalcohols

## Abstract

Oxylipins are one of the most important classes of bioregulators, biosynthesized through the oxidative metabolism of unsaturated fatty acids in various aerobic organisms. Oxylipins are bioregulators that maintain homeostasis at the cellular and organismal levels. The most important oxylipins are mammalian eicosanoids and plant octadecanoids. In plants, the main source of oxylipins is the lipoxygenase cascade, the key enzymes of which are nonclassical cytochromes P450 of the CYP74 family, namely allene oxide synthases (AOSs), hydroperoxide lyases (HPLs), and divinyl ether synthases (DESs). The most well-studied plant oxylipins are jasmonates (AOS products) and traumatin and green leaf volatiles (HPL products), whereas other oxylipins remain outside of the focus of researchers’ attention. Among them, there is a large group of epoxy hydroxy fatty acids (epoxyalcohols), whose biosynthesis has remained unclear for a long time. In 2008, the first epoxyalcohol synthase of lancelet *Branchiostoma floridae*, BfEAS (CYP440A1), was discovered. The present review collects data on EASs discovered after BfEAS and enzymes exhibiting EAS activity along with other catalytic activities. This review also presents the results of a study on the evolutionary processes possibly occurring within the P450 superfamily as a whole.

## 1. Introduction

**Figure 1:**
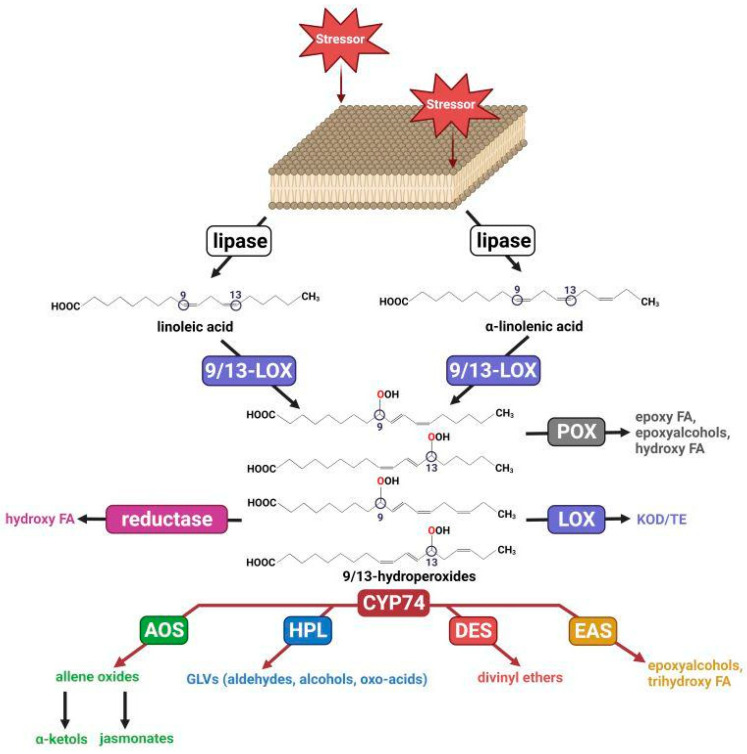
Scheme of lipoxygenase cascade. LOX—lipoxygenase; POX—peroxygenase; AOS—allene oxide synthase; HPL—hydroperoxide lyase; DES—divinyl ether synthase; EAS—epoxyalcohol synthase; FA—fatty acid; KOD/TE—ketooctadecadi(tri)enoic acid. This figure was created with BioRender.com (accessed on 28 November 2023).

The oxidation of polyunsaturated fatty acids (PUFAs) is a source of oxylipins, important bioregulators which play a significant role in regulatory processes, as well as responses to changes in environmental conditions [1,2,3,4,5,6,7,8]. For example, in mammals, eicosanoids, products of oxidative transformations of eicosane fatty acids, control the functioning of the digestive organs, the cardiovascular and respiratory systems, and the reproduction system, and participate in inflammatory processes, anaphylaxis, immune response systems, etc. [9,10,11,12,13,14,15,16]. The oxidative metabolism of PUFAs in plants is much less widely studied. The main source of oxylipins in plants is the lipoxygenase cascade, which starts with the formation of fatty acid hydroperoxides by lipoxygenases (Figure 1). The further metabolism of hydroperoxides is controlled by a number of enzymes, including non-classical cytochromes P450 of the CYP74 family [2]. The most common CYP74 enzymes are allene oxide synthase (AOS) and hydroperoxide lyase (HPL, synonym “hemiacetals synthase” [17]). They were found in all flowering plants studied to date. Much less common are divinyl ether synthase (DES), which are found in several plant species phylogenetically distant from each other [18,19,20,21].

Classical P450s include monooxygenases, which catalyze electron transfer from NAD(P)H to molecular oxygen and the regio- and stereospecific incorporation of an oxygen atom into the substrate [22,23]. In contrast, the CYP74 enzymes require neither molecular oxygen nor external electron donors for their catalytic activity [22,24,25,26]. They use fatty acid hydroperoxides, which serve both as a substrate and an oxygen donor. In animals, analogues of CYP74 enzymes can be considered, for example, prostacyclin synthase (PGI2, CYP8A1) and thromboxane synthase (TXA2, CYP5A1) in mammals, which convert prostaglandin endoperoxides [25,27,28].

Recently, enzymes similar to members of the CYP74 family, as well as oxylipins similar to the products of CYP74s, have been identified in taxonomically distant organisms, including proteobacteria and Metazoa [25], as well as brown [29] and red [30] algae. According to the requirements of the nomenclature (more than 40% amino acid sequence identity), the identified enzymes cannot be attributed to the CYP74 family; therefore, the concept of the CYP74 clan was introduced [31]. The CYP74 clan includes enzymes of the CYP74 family as well as other families that are similar to CYP74s in structure, their catalytic mechanisms, and the results of phylogenetic studies.

There are oxylipins that are formed enzymatically or spontaneously (phytoprostanes and isoprostanes). Phytoprostanes are currently receiving much attention due to their structural similarity to biological response mediators in mammals, such as isoprostanes and prostanoids [32,33,34]. In recent years, the diversity of phytoprostanes in various plant species and products, including vegetable oils, seeds, and non-edible plant materials, has been actively studied. They are considered as biomarkers of the physiological state of plants [35,36,37] and the oxidative degradation of food products of plant origin [38], and also as bioactive compounds that have anti-inflammatory and immunomodulatory properties, including preventing neuronal damage in humans [32,39,40,41].

In flowering plants, there are different groups of oxylipins, products of the lipoxygenase cascade, namely hydroxy-, dihydroxy-, trihydroxy-, oxo-, epoxy-, and keto-derivatives of fatty acids, divinyl ethers, aldehydes, alcohols, aldoacids, cyclopentenones, and jasmonates [7,42,43,44,45,46]. Additionally, unusual oxylipins, such as graminoxins [47], and complex oxylipins, such as linolipins [48], have been identified. The physiological properties of plant oxylipins have been studied, with unjustifiably great attention being paid to jasmonates [49,50,51,52,53,54,55,56,57,58,59,60,61,62,63], traumatin, and green leaf volatiles (GLVs) [46,64,65,66,67,68,69,70,71,72,73,74,75,76,77,78]. Much less attention has been paid to other branches of the lipoxygenase cascade, including the formation of epoxy hydroxy derivatives (epoxyalcohols) and trihydroxy derivatives (trihydroxy acids), despite the fact that these compounds are found in organisms belonging to different taxa [79,80,81,82,83,84,85,86,87,88,89,90,91,92].

Two different mechanisms are known for the conversion of fatty acid hydroperoxides to epoxyalcohols. The first mechanism occurs through the reduction of the peroxy moiety and the epoxidation of one double bond. Such reactions are catalyzed by peroxygenases [93,94,95] and other oxidoreductases [96]. The second mechanism occurs through homolysis of the O-O bond of the hydroperoxide, rearrangement of the oxy radical into an epoxyallyl radical, and reduction of the hydroxyl radical. This mechanism has been shown to occur in the presence of acids [97,98], Fe^3+^ ions [99,100,101], hemoproteins [102], and upon heating [103]. Epoxyalcohol production from hydroperoxides in the presence of monooxygenases [104] has been observed, but at a low yield and a much slower rate compared to the EsEAS. Epoxyalcohols have also been detected as the minor side products of some CYP74 enzymes, in particular, the AOSs [105,106], as well as some nonclassical P450 AOSs of fungi [107,108].

In 2008, the first enzyme catalyzing the formation of epoxyalcohols via the second mechanism was discovered, namely epoxyalcohol synthase (EAS) BfEAS (CYP440A1) from the lancelet *Branchiostoma floridae* Hubbs, 1922 [25]. This enzyme belongs to the CYP74 clan, suggesting that at least some of the EASs will belong to the CYP74 clan or family. However, despite the significant interest of researchers in CYP74 enzymes, BfEAS (CYP440A1) is still the only representative of this group of enzymes. This group appears to be widespread, based on the fact that products of the EAS reaction have been found in many species, e.g., *Bryonia alba* and *Nicotiana tabacum* [109,110,111]. Thus, the main goal of the work was to identify the enzymes involved in the biosynthesis of epoxyalcohols, products of EAS activity.

## 2. Epoxyalcohol Synthase Activity in Different CYP74 Enzymes

As a result of our studies, it was shown that the enzymes previously described and annotated as 9/13-specific HPL, namely CYP74C1_CS (*Cucumis sativus*), CYP74C2 (*Cucumis melo*), CYP74C4_ST (*Solanum tuberosum*), CYP74C13_GM (*Glycine max*), CYP74C13_MT (*Medicago truncatula*), and CYP74C31 (*C. sativus*), are enzymes with double HPL/EAS activity [112]. The results of the incubation of these enzymes with hydroperoxides were as follows: the conversion of 9-hydroperoxides led to the formation of 9-hydroxynonanoic acid, while the conversion of 13-hydroperoxides led to the formation of isomers of 12-hydroxydodecenoic acid. These are hydroperoxide lyase (HPL) products [17]. In addition, the conversion of linoleate 9-hydroperoxide (9-HPOD) led to the formation of 9,10-epoxy-11-hydroxy-12-octadecenoic and 9,10-epoxy-13-hydroxy-11-octadecenoic acids. The conversion of α-linolenate 9-hydroperoxide (9-HPOT) led to the formation of 9,10-epoxy-11-hydroxy-12,15-octadecadienoic acid. The conversion of linoleate 13-hydroperoxide (13-HPOD) led to the formation of 11-hydroxy-12,13-epoxy-9-octadecenoic acid. These are epoxyalcohols, products of EAS activity. Thus, along with HPL activity, these enzymes possessed EAS activity. Moreover, the following trend was observed in all of the described enzymes: in the series 9-HPOD, 9-HPOT, 13-HPOD, and α-linolenate 13-hydroperoxide (13-HPOT), EAS activity decreases while HPL activity increases. Thus, since the rates of reactions catalyzed by these enzymes towards all substrates were nearly the same, one can conclude that these six enzymes possess double HPL/EAS activity. However, not all enzymes previously described or annotated as 9/13-HPLs possess double HPL/EAS activity. We discovered CYP74C43 (NtHPL of tobacco (*Nicotiana tabacum*)), which is an HPL with additional EAS activity [111], and CYP74C44 (ShHPL of the neotropical fruit bat *Sturnira hondurensis*) which is a true HPL [113]. Moreover, ShHPL is the only CYP74 enzyme detected in mammals.

After discovering EAS activity in CYP74C HPLs, the next stage was checking for EAS activity in other CYP74s—firstly, CYP74A and CYP74C AOSs. Allene oxide synthase LeAOS3 (CYP74C3) of tomato (*Solanum lycopersicum*) belongs to the same CYP74C subfamily, as CYP74 enzymes with double HPL/EAS activity. This AOS possesses multifunctional activity, catalyzing not only the formation of allene oxide but also its hydrolysis and cyclization [114]. However, there is no EAS activity in LeAOS3. In addition, CYP74A AOSs only catalyze the formation of allene oxides [115]. No additional activities were detected [116].

The CYP74C subfamily includes enzymes possessing 9/13-HPL activity. At the same time, 13-specific HPLs belong to the CYP74B subfamily. We checked EAS activity in three CYP74B enzymes, namely StHPL (CYP74B3) of *S. tuberosum*, MsHPL (CYP74B4) of *Medicago sativa*, and CsHPL (CYP74B6) of *C. sativus*. These enzymes possess the main HPL and minor EAS activity towards the preferred substrates, 13-hydroperoxides. On the other hand, these enzymes possess the main EAS and minor HPL activity towards the non-preferred substrates, 9-hydroperoxides. Thus, the CYP74B enzymes are 13-specific HPLs possessing additional EAS activity [117].

In addition, the following trend was observed in the transformation of 13-hydroperoxides: the conversion of 13(*S*)-hydroperoxide of α-linolenic acid occurs more specifically than the conversion of 13(*S*)-hydroperoxide of linoleic acid. In the second case, more epoxyalcohols are produced than in the first one. However, in both cases, HPL products are the major ones. Apparently, the presence of a ω3 double bond favors the HPL type of catalysis. The ω3 double bond can affect the conversion of the epoxyallylic radical intermediate due to the assistance of the C-C bond opening within the oxirane of the epoxyallylic radical. The oxirane opening leads to the vinyloxycarbinyl radical, which is then recombined with the hydroxyl radical to form the hemiacetal, an immediate HPL product. In the absence of a ω3 double bond, recombination of the epoxyallylic radical with the hydroxyl radical occurs more often, leading to the formation of an epoxyalcohol, the EAS product [117]. The schemes of HPL and EAS reactions are presented below.

The CYP74B subfamily does not solely include 13-specific HPLs. The CYP74B33 enzyme of *Daucus carota* possesses AOS activity towards preferred substrates, 9-hydroperoxides. At the same time, this enzyme possesses mixed AOS, HPL, and EAS activities towards non-preferred substrates, 13-hydroperoxides [118]. Moreover, the CYP74B16 enzyme of *Linum usitatissimum* possesses double DES/HPL activity with additional EAS activity [119].

After checking the presence of EAS activity in HPLs and AOSs, the next targets were CYP74D DESs. These enzymes behave as DESs with additional minor HPL and EAS activities towards preferred substrates, 9-hydroperoxides. 13-Hydroperoxides are poor substrates for these enzymes [119].

The CYP74D DESs catalyze the formation of colneleic and colnelenic acids from 9-hydroperoxides. At the same time, there are several plant species in which divinyl ethers, derivatives of 13-hydroperoxides, were detected. We discovered 13-specific DESs in *Ranunculus acris* and *Selaginella moellendorffii*. These enzymes catalyze the formation of different isomers of etheroleic and etherolenic acids. *R. acris* CYP74Q1 yields (ω5*Z*)-isomers [120]. The CYP74M1 of *S. moellendorffii* forms (11*Z*)-isomers as the main products and etheroleic and etherolenic acids as minor products. Alternatively, CYP74M3 of *S. moellendorffii* yields etheroleic and etherolenic acids as the main products and their (11Z)-isomers as minor products [121]. Additionally, (ω5*Z*)-isomers are minor products for CYP74M1 and CYP74M3. All three enzymes are true DESs. No AOS, HPL, or EAS products were detected. In addition to the CYP74D subfamily of 9-specific DESs, as well as the CYP74Q and CYP74M subfamilies of 13-specific DESs, there is the CYP74H subfamily, including 9/13-specific DESs of garlic (AsDES, CYP74H1) [122] and asparagus (AoDES, CYP74H2) [123]. These enzymes are also true DESs.

The CYP74M2 enzyme of *S. moellendorffii* possesses EAS activity, forming three isomers of 11-hydroxy-12,13-epoxy-9-octadecenoic acid from 13-HPOD, 11-hydroxy-12,13-epoxy-9,15-octadecadienoic acid from 13-HPOT, and 9,10-epoxy-11-hydroxy-12-octadecenoic acid from 9-HPOD. In the last case, trace AOS and HPL products were detected. However, due to specific EAS activity towards preferred substrates, 13-hydroperoxides, CYP74M2 is the first true EAS found in planta belonging to the CYP74 family [124]. The second true plant EAS was detected in *Ranunculus japonicus*. Conversion of linoleate hydroperoxides by RjEAS (CYP74A88) leads to the formation of epoxyalcohols [125].

All of the enzymes listed above have been studied as recombinant proteins obtained in *E. coli* and purified using metal affinity chromatography. The open reading frames of the corresponding genes were cloned or commercially synthesized. The products of enzymes were studied using HPLC, GC-MS, and NMR. Almost all CYP74 enzymes have a neutral pH optimum for catalytic activity (7 or 7.5). The exception is the CYP74B enzymes. StHPL (CYP74B3) and MsHPL (CYP74B4v1) have their pH optima at 8.0, while the pH optimum for CsHPL (CYP74B6) was found at 6.0. The pH optima for the catalytic activities of the StHPL, MsHPL, and CsHPL indirectly indicate the subcellular localization of these enzymes. It was shown that the CYP74B 13-specific HPLs are localized in the outer envelope of chloroplast, with most of the protein being exposed to the inter-membrane space [126]. The inter-membrane space of chloroplast has a mean pH 6.0, whereas the stroma has a mean pH 8.0. Thus, the CsHPL enzyme seems to have an inter-membrane localization, while StHPL and MsHPL have a stromal localization. The localization of the MsHPL enzyme is confirmed by the presence of chloroplastic transit peptide in this sequence [65].

The first EAS was discovered in the lancelet *B. floridae* [25]. Thus, EASs are apparently an ancient group of enzymes that appeared before the separation of plants and Metazoa. We identified and cloned the genes of four CYP74-like proteins belonging to the CYP74 clan (not family) from non-plant organisms, namely the brown alga *Ectocarpus siliculosus* [127], the starlet sea anemone *Nematostella vectensis* [128,129], and the lancelet *Branchiostoma belcheri* [130]. CYP5164B1 of *E. siliculosus* [127] and CYP443D1 of *N. vectensis* [128] are true EASs, while two other enzymes possess double activities. However, recently, CYP5164B1 and novel enzymes, namely hydroperoxide bicyclases [131,132], were united into a separate clan, CYP5164. CYP443C1 of *N. vectensis* [129] and the CYP440A18 [130] enzymes possess double HPL/EAS and EAS/AOS activities, respectively.

In order to shed light on the EAS catalytic mechanism, experiments were carried out using ^18^O. The results indicated that EASs are isomerases. The first stage of the EAS reaction is the homolysis of the hydroperoxy moiety resulting in the formation of an alkoxy radical, which is rearranged into an epoxyallyl radical. In the last step, the epoxyallyl radical recombines with the hydroxyl radical to form an epoxyalcohol [127,128].

The comparison of the structures of products synthesized by different EASs revealed that plant EASs, as well as EsEAS, mainly synthesize (9*S*,10*S*,11*S*)-epimer with *trans*-epoxide, while metazoan EASs produce (*S*,*R*,*S*)-stereoisomers with *cis*-epoxide [125,126,127,128].

## 3. Phylogeny

**Figure 2:**
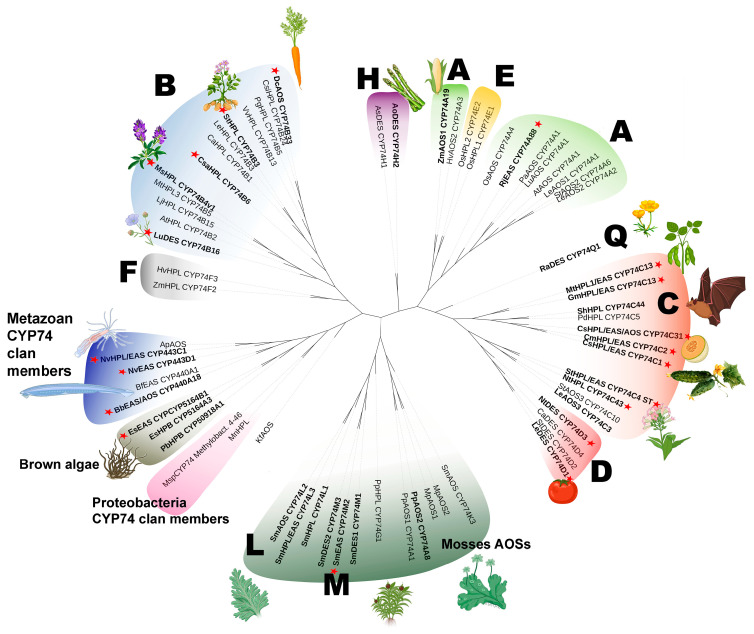
The unrooted phylogenetic tree of the CYP74 clan. Classified CYP74 subfamilies are marked with their letter designation (A, B, C, etc.). Subfamilies consisting of more than one member are outlined with unclosed curves (semi-ellipses). Enzymes exhibiting EAS activity are indicated in bold. True EASs are marked with red asterisks. The Plant CYP74s: As, *Allium sativum* AsDES, CYP74H1, GI: 83414021; Ao; *Asparagus officinalis*, AoDES, GI: 109845459; At, *Arabidopsis thaliana*; AtAOS, CYP74A1, GI: 15239032; AtHPL, CYP74B2, GI: 3822403; Ca, *Capsicum annuum*; CaHPL, CYP74B1, GI: 1272340, CaDES, CYP74D4, GI: 107840369; Cm, *C. melo*; CmHPL/EAS, CYP74C2, GI: 14134199; Csa, *C. sativus*; CsaHPL/EAS, CYP74C1_CS, GI: 101211324; CsaHPL/EAS/AOS, CYP74C31 GI: 101211574; CsaHPL, CYP74B6, GI: 101223126; Csi*, Camellia sinensis;* CsiHPL, CYP74B24, SI: BAU24783.1; Dc, *D. carota;* CYP74B33, GI: 108219710; Hv, *Hordeum vulgare;* HvAOS2, CYP74A3, SI: AJ251304.1; HvHPL, CYP74F3, CAC82980.1; Kf, *Klebsormidium flaccidum* (green alga); KfAOS, SI: LC032459.1; Le, *S. lycopersicum;* LeAOS1, CYP74A1, GI: 7581989; LeAOS2, CYP74A2, GI: 7677376; LeAOS3, CYP74C3, GI: 25991603; LeHPL, CYP74B3, GI: 7677378 LeDES, CYP74D4, GI: 543675; Lj, *Lotus japonicus;* LjHPL, CYP74B15, SI: AB600748.1; Lu, *L. usitatissimum;* LuAOS, CYP74A1, GI: 1352186; LuDES, CYP74B16, GI: 379048766; Mp, *Marchantia polymorpha*; MpAOS1, SI: LC032457.1, MpAOS2, SI: LC032458.1; Mt, *M. truncatula*; MtHPL3, CYP74B5, GI: 63081244; MtHPL1/EAS, CYP74C13_MT, GI: 33504430; Nt, *N. tabacum*; NtDES, CYP74D3; GI: 107799697; Os, *Oryza sativa*; OsAOS, CYP74A4, GI: 115455571; OsHPL1, CYP74E2, GI: 115445057; OsHPL2, CYP74E1, GI: 125538638; Pa, *Parthenium argentatum;* PaAOS, CYP74A1, GI: 218511958; Pd, *Prunus dulcis;* PdHPL, CYP74C5, GI: 33300600; Pg, *Psidium guajava*; PgHPL, CYP74B5, GI: 13183137; Pp, *Physcomitrella patens*; PpAOS1, CYP74A1, GI: 22217985; PpAOS2, CYP74A8, GI: 168014176; PpHPL, CYP74G1, GI: 76057841; Ra, *R. acris;* RaDES, CYP74Q1, GI: 768564485; Rj, *R. japonicus*; RjEAS, CYP74A88, SI: MK061531; Sm, *S. moellendorffii*; SmDES1, CYP74M1, GI: 9660714; SmEAS, CYP74M2, GI: 9637471; SmDES2, CYP74M3, GI: 9654395; SmHPL, CYP74L1, GI: 9645914; SmAOS, CYP74L2, GI: 9651730; CYP74L3, SI: EFJ25870; SmAOS, CYP74K3, SI: EFJ20163.1; St, *S. tuberosum;* StAOS2, CYP74A6, GI: 86769479; StAOS3, CYP74C10, GI: 56605358; StHPL/EAS, CYP74C4, GI: 102588560; StDES, CYP74D2, GI: 12667099; Vv, *Vitis vinifera*; VvHPL, CYP74B13, FJ861082; Zm, *Zea mays*; ZmAOS, CYP74A19, GI: 223947589; ZmHPL, CYP74F2, GI: 162462890. CYP74 clan members: Es, *E. siliculosus* (brown alga); EsEAS, CYP5164B1, GI: 1109557544; EsHPB, CAB1111511.1; Mn, *Methylobacterium nodulans* (proteobacteria); MnHPL, SI: WP_015932840.1; Msp, Methylobacterium sp. 4–46; MspCYP74, SI: WP_012335549.1. Ap, *Acropora palmata* (Metazoa); ApAOS, GI: 187948710; Bf, *B. floridae* (Metazoa); BfEAS, CYP440A1, GI: 189312561; Bb, *B. belcheri*; BbEAS/AOS, XP_019641998.1; Nv, *N. vectensis* (Metazoa); NvEAS CYP443D1, GI: 5516222; NvHPL/EAS CYP443C1; Pb, *Plasmodiophora brassicae; PbHBP*, CYP50918A1, CEO97746.1. The tree is drawn to scale, with branch lengths measured as the number of substitutions per site. The evolutionary history was inferred by using the Maximum Likelihood method (1000 replicates). Evolutionary analyses were conducted in MEGA7. The iTOL tool version 6.8.1 (https://itol.embl.de/) was used to visualize the phylogenetic model output. This figure was created with BioRender.com (accessed on 1 December 2023).

The initial classification of CYP74 enzymes was as follows: the CYP74A and CYP74B subfamilies included 13-specific AOSs and HPLs, respectively; the CYP74C subfamily included 9- and 9/13-specific AOSs and HPLs; and the CYP74D subfamily included 9-specific DESs. We constructed a phylogenetic tree on the basis of the results obtained by us and other researchers (Figure 2). As a result of recent research, it was shown that 13-specific EASs were also found in the CYP74A subfamily; a 13-specific enzyme with double DES/HPL activity and a 9-specific AOS were described in the CYP74B subfamily along with 13-specific HPLs. Additionally, the CYP74C enzymes, previously characterized or annotated as 9/13-specific HPLs, have been shown to be enzymes with double HPL/EAS activities. In addition, members of the new CYP74J, CYP74K, CYP74L, CYP74M, and CYP74Q subfamilies, as well as members of the CYP74 clan but not the family, were identified and characterized, namely *E. siliculosus* and *N. vectensis* EASs, as well as the *N. vectensis* enzyme with double HPL/EAS activity and the *B. belcheri* enzyme with double EAS/AOS activity.

On the phylogenetic tree, true EASs, as well as enzymes exhibiting EAS activity, are distributed evenly throughout the tree and are not clustered in one area. This may indicate that they all have either ancient or independent origins.

## 4. Correlation between Primary Structure and Catalytic Activity of CYP74s

**Figure 3:**
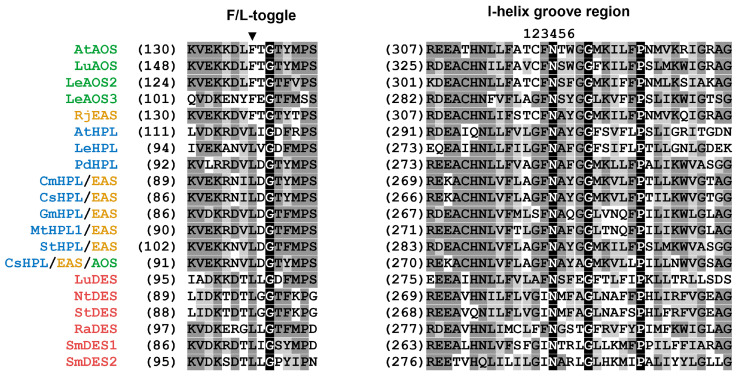
Multiple alignment of partial sequences of CYP74s: SRS-1 (**left**) and I-helix (**right**). The I-helix groove region (SRS-4) is numbered 1–6, the first glycine residue after this domain is marked with a ▼ symbol, and the F/L toggle site (SRS-1) is marked with an asterisk. The following CYP74 sequences were used for alignment: At, *A. thaliana*; AtAOS, NP_199079.1, AtHPL, Q9ZSY9.1; Cs, *C. sativus*; CsHPL/EAS, CYP74C1_CS, NP_001274399.1, CsHPL/EAS/AOS, CYP74C31, XP_004137005.1; Cm, *C. melo*; CmHPL/EAS, CYP74C2, NP_001284390.1; Gm, *G. max*; GmHPL/EAS, CYP74C13_GM, XP_028186824.1; Le, *S. lycopersicum*; LeAOS2, NP_001274707.1; LeAOS3, NP_001265949.1, LeHPL, AAF67142.1; Lu, *L. usitatissimum*; LuDES, ADP03054.2, LuAOS CYP74A1 P48417.1; Mt., *M. truncatula*; MtHPL/EAS, CYP74C13_MT, XP_003606860.1; Nt, *N. tabacum*; NtDES CYP74D3 NP_001312606.1; *R. japonicus*; RjEAS, CYP74A88, QCR70269.1; St, *S. tuberosum*; StHPL/EAS, CYP74C4_ST, XP_006365486.1; Sm, *S. moellendorffii*; SmDES1, CYP74M1, XP_002979266.1; SmEAS, CYP74M2 XP_002964012.2. Alignment was performed using Vector NTI 11 program (Invitrogene, Madison, WI, USA).

Alignment of the amino acid sequences of biochemically characterized CYP74 enzymes suggested a correlation between amino acid residues in individual sites (mainly substrate recognition sites, SRSs [133]) and the type of catalytic activity. The first determinant of CYP74 catalysis, the F/L toggle site, is located in SRS-1 near the N-terminus. All AOSs and two described plant EASs (RjEAS and SmEAS) contain a phenylalanine residue at this site, while all known HPLs and DESs contain a leucine residue. In SRS-4, there is the I-helix groove region (earlier hydroperoxide-binding domain [112]) corresponding to the oxygen-binding domain of monooxygenases P450. The CYP74 enzymes require neither molecular oxygen nor redox partners; thus, the structure of their catalytically essential domains differs from that of monooxygenases P450. As a whole, the I-helix groove region has a sequence that is conservative for a particular type of catalysis (Figure 3). To determine the interrelation between the primary structure and the catalytic activity of CYP74 enzymes, the method of site-directed mutagenesis was chosen. A number of mutants were obtained, and their catalytic properties were analyzed. The following results were obtained.

Three mutants of three CYP74C enzymes possessing double HPL/EAS activity, namely CYP74C1_CS, CYP74C13_MT, and CYP74C31, were obtained. Mutations at the F/L toggle, at the I-helix groove region, and especially combinations of these mutations led to the appearance of AOS activity along with a simultaneous decrease in EAS and HPL activities [112]. On the contrary, substitution at the F/L toggle in the tomato LeAOS3 sequence led to the appearance of EAS activity and a decrease in AOS activity [134].

A similar substitution at the F/L toggle was carried out in the sequences of AOSs of the moss *P. patens* (PpAOS2, CYP74A1) and maize (ZmAOS1, CYP74A19). The mutant form of PpAOS2 lost its AOS activity and acquired EAS and HPL activities towards 9-hydroperoxides. The mutant form of ZmAOS1 retained its AOS activity towards 9-hydroperoxides, while additional EAS and HPL activities appeared. Towards 13-hydroperoxides, the mutant forms of both enzymes retained their AOS activity; HPL and EAS activities also appeared. In the case of these mutants, analogously to the wild-type CYP74C enzymes with double HPL/EAS activity, HPL activity was more expressed towards 13-hydroperoxides, and EAS activity was more expressed towards 9-hydroperoxides [116].

In addition to the F/L toggle site and the I-helix groove region, another catalytically essential domain is the ERR triad. Substitutions at the I-helix groove region and the ERR triad in the LeAOS3 sequence resulted in an alteration to AOS-to-HPL activity [135]. At the same time, substitutions in the same domains in the sequence of flax CYP74B16 enzyme with double DES/HPL activity and tobacco DES CYP74D3 (NtDES) led to the conversion of these enzymes into AOSs [136]. However, mutations in other sites of the I-helix groove region in the sequence of the CYP74B16 led to a shift in the activity of this enzyme from DES to HPL and EAS [119].

**Figure 4:**
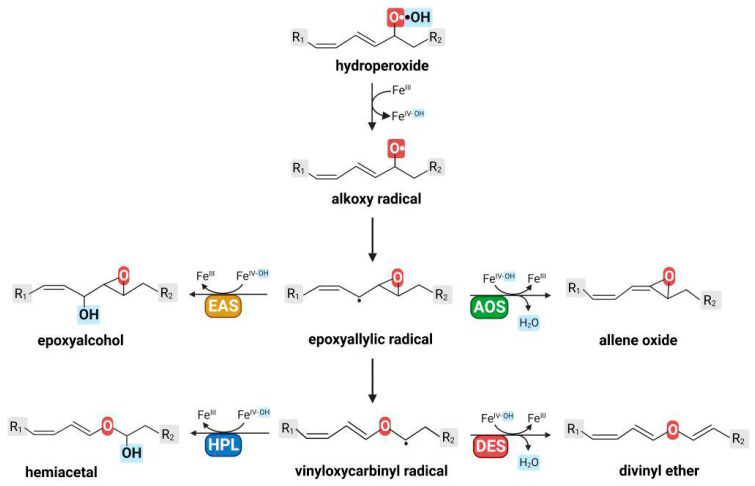
Scheme of CYP74 catalytic mechanisms. This figure was created with BioRender.com (accessed on 28 November 2023).

The results of site-directed mutagenesis experiments indicated a close relationship between different types of catalysis (Figure 4). Two initial steps of catalysis are common to all CYP74 enzymes. In the first stage, the peroxy moiety undergoes homolytic cleavage to form an alkoxy radical intermediate, which rearranges into an epoxyallylic radical. The epoxyallylic radical is a common intermediate of all CYP74 enzymes and a switch point between all four catalytic mechanisms. Depending on the CYP74 primary structure, the epoxyallylic radical undergoes either (1) oxidation followed by proton loss to form the allene oxide (AOS pathway) or (2) recombination with hydroxyl radical to form the epoxyalcohol (EAS pathway), or undergoes homolytic opening of the oxirane to form a vinyloxycarbinyl radical, which is the switch point between HPL and DES catalytic activities. The further stages are similar to those of the EAS and AOS mechanisms. In the case of the HPL pathway, a vinyloxycarbinyl radical recombines with a hydroxyl radical to afford the hemiacetal; in the case of the DES pathway, it is oxidized and loses a proton to form divinyl ether.

**Figure 5:**
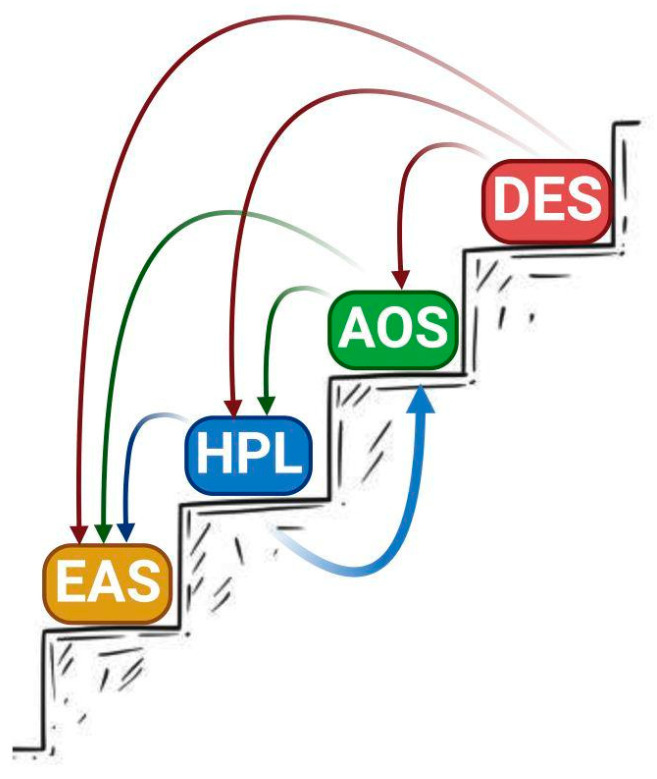
Scheme of the “straightway” of the evolution of CYP74 reactions. This figure was created with BioRender.com (accessed on 28 November 2023).

Qualitative changes in CYP74 catalysis, namely AOS into HPL or EAS, and enzyme with double HPL/EAS activity into AOS, and DES into AOS, HPL, or EAS have suggested evolution within the CYP74 family (Figure 5). So, the evolution of CYP74 reactions apparently followed the path of EAS–HPL–AOS–DES. From the point of view of molecular evolution, the EAS reaction is probably the basic one, and HPL, AOS, and DES reactions were built up as a result of modifying this basic reaction due to the additional influence of side groups of new amino acids that appeared during evolution and point mutations. Thus, novel enzymes have appeared as a result of duplication and mutation. At the present time, one can observe this process in the genome of some species [137]. The results of site-directed mutagenesis are representative of the opposite processes, reversions. As a result of each substitution, there is a kind of step down in the evolutionary “straightway”. As a result of several simultaneous substitutions, we were able to simulate the rise from the level of HPL to the level of AOS.

Some of the resulting mutants had two or even three activities. Wild-type enzymes can also possess two or even three activities. A possible explanation for this phenomenon may lie in the specificity of cytochromes P450 in general. Many cytochromes P450 exhibit conformational plasticity, which allows them to adapt to different substrates [138,139]. And probably, for some cytochromes P450 as a whole, the utilization of the substrate is a more important event than the formation of the product. The organism may be influenced by more and more potentially harmful or dangerous molecules, possible P450 substrates. The formation of new P450 enzymes cannot proceed at the same rate, so it should be a possibility to utilize these molecules. Currently, this property is used to redesign enzymes for specific reactions through rational design [140]. This hypothesis is also suitable to CYP74 enzymes.

The P450 superfamily is one of the largest superfamilies of enzymes, demonstrating special patterns of evolution, including bursts of diversification [141]. It is most probable that all cytochrome P450s have a common ancestor and evolved into different forms with different activities [142]. The appearance of the ancient P450s is apparently associated with the accumulation of molecular oxygen in the atmosphere, resulting in the necessity to protect the cell from biomolecules oxidized by reactive oxygen species. These ancient P450s, in general, could use the only substrate, the product of spontaneous oxidation of biopolymers, without additional oxygen or redox partners. It is possible that the CYP74 enzymes that catalyze a similar reaction towards fatty acid hydroperoxides are vestigial representatives or direct descendants of this ancient group of oxygen-independent cytochromes P450. CYP74s catalyze the conversion of oxidized fatty acids included in the membrane into different diffusible and volatile products. The anchoring of these enzymes in evolution may have occurred due to the fact that one of the products was integrated into the metabolism and provided a serious advantage for the organism. In addition, the further evolution process consisted not of the specialization of the enzyme in the synthesis of this particular compound [143], but of the integration of other products into metabolism, which provided additional advantages.

**Figure 6:**
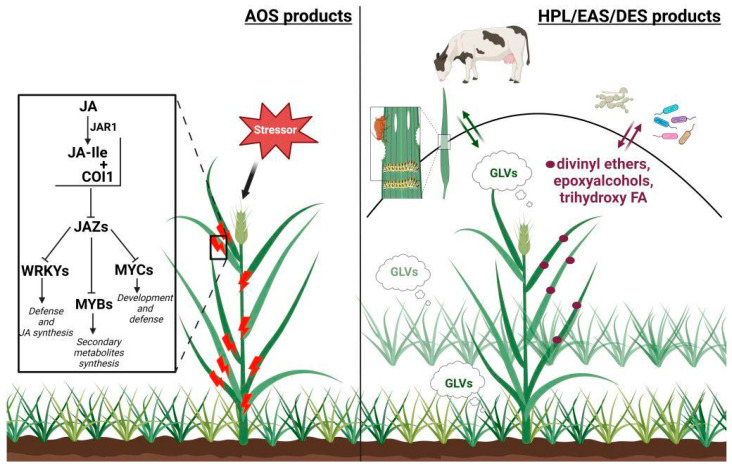
Differences between two functional groups of CYP74 enzymes: “signaling” and “communicative”. JA—jasmonic acid; JA-Ile—jasmonoyl-L-isoleucine; COI—coronatine insensitive1 protein; JAZ—jasmonate ZIM-domain protein; WRKY—WRKY transcription factor; MYB—MYB transcription factor; MYC—MYC transcription factor. This figure was created with BioRender.com (accessed on 27 November 2023).

Currently, the CYP74 enzymes can be divided into two groups: “signaling” and “communicative” (Figure 6). AOSs can be separated from others. They are usually specific enzymes that convert hydroperoxides into allene oxides. Some AOSs catalyze not only the biosynthesis of allene oxides but also their hydrolysis and cyclization. However, the final products, namely jasmonates, cyclopentenones, and ketols, are signaling compounds that generally work inside the plant [2,50,52,57,58,59,61,144,145,146,147]. Thus, AOSs may be determined as a “signaling” group. Alternatively, HPL and EAS activities are mostly combined in the same enzyme. These activities yield different products involved in plant communication. Thus, GLVs (HPL products) are compounds of communication between plants and other organisms (plants, insects, herbivores) [67,70,71,144,148], whereas epoxyalcohols (EAS products) possess antimicrobial and fungicide properties [45,149]. The last group of enzymes, DESs, is apparently the last of the described evolutional acquisitions of plants. DESs produce antimicrobial and fungicide compounds, divinyl ethers [20,45,150,151]. DESs are detected in several plant species that are phylogenetically distant from each other. Some of them are highly specific enzymes. However, even if DES activity is possessed along with that of other enzymes, it combines with HPL and/or EAS activities, since DESs, like HPLs and EASs, belong to the “communicative” group, not “signaling”.

**Figure 7:**
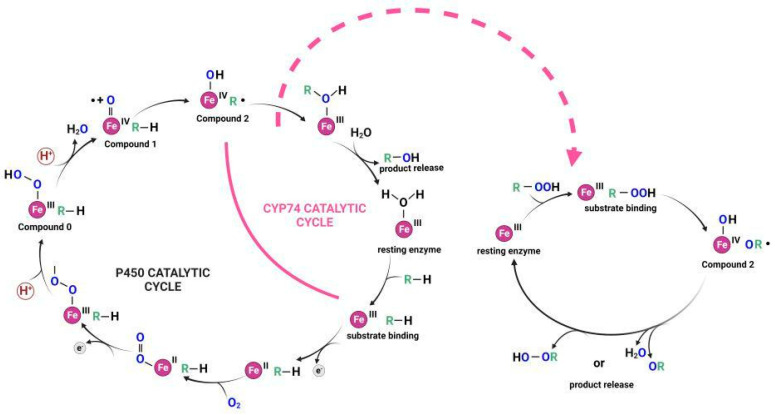
The scheme of catalytic cycles of classical cytochromes P450 and CYP74 enzymes. This figure was created with BioRender.com (accessed on 27 November 2023).

The ancient origin of CYP74s is confirmed by their structural features, catalytic mechanisms, and the results of phylogenetic studies. Almost all cytochromes P450 are monooxygenases, and their catalytic action requires molecular oxygen, a redox partner, and a substrate. A unique feature of all CYP74s is the lack of a requirement for molecular oxygen and redox potentials. Fatty acid hydroperoxide is a source of both. As a result, the catalytic cycle of CYP74s is simpler compared to that of classical cytochromes P450 (Figure 7).

Structural features also confirm the hypothesis that the structure of some CYP74s is similar to that of catalases [152], enzymes catalyzing the decomposition of hydrogen peroxide into water and oxygen. In addition, in marine Metazoa, there are a number of enzymes that catalyze AOS and HPL reactions and are not cytochromes P450, but the catalase-related haemoproteins, for instance, the AOSs of soft corals *Plexaura homomalla* [153], *Gersemia fruticosa* [154], *Capnella imbricata* [155], *Acaryochloris marina* [156], as well as the catalase-type HPL of soft coral *Capnella imbricata* [157] (see [158] for a review). The intronless structure of the majority of CYP74 genes also indirectly confirms the ancient origin of CYP74s.

**Figure 8:**
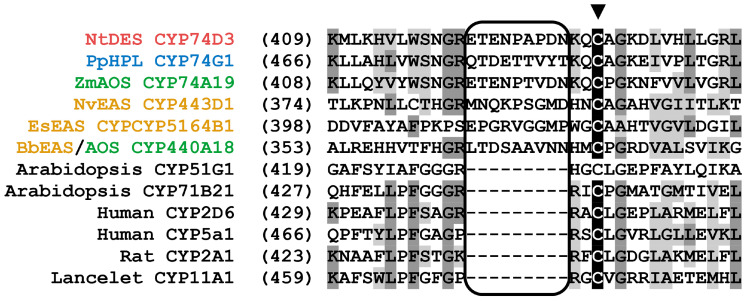
The P450s sequences alignments. The CYP74 family sequences: Nt, *N. tabacum*; NtDES CYP74D3 NP_001312606.1; Pp, *P. patens*; PpHPL, CYP74G1, XP_024376101.1; Zm, *Z. mays*; ZmAOS, CYP74A19, ACG28578.1; Nv, *N. vectensis* (Metazoa); NvEAS CYP443D1, ASS83181.1; Es, E*. siliculosus* (brown alga); EsEAS, CYP5164B1, A0A1L3HS58.1; Bb, *B. belcheri* BbEAS/AOS CYP440A18, XP_019641998.1 The P450 monooxygenases sequences: At, *A. thaliana*; CYP51G1 NP_172633.1, CYP71B21 KAG7626632.1; *Homo sapiens* (Human) CYP2D6 ABB01371.1, CYP5a1 AAF99279.1; *Rattus norvegicus* (Rat) CYP2A1 P11711.2; *B. lanceolatum* (lancelet) CYP11A1 CAH1250563.1. Alignment was performed using Vector NTI 11 program (Invitrogene, USA). The conservative haem-binding cysteine residue is marked with the triangle.

**Figure 9:**
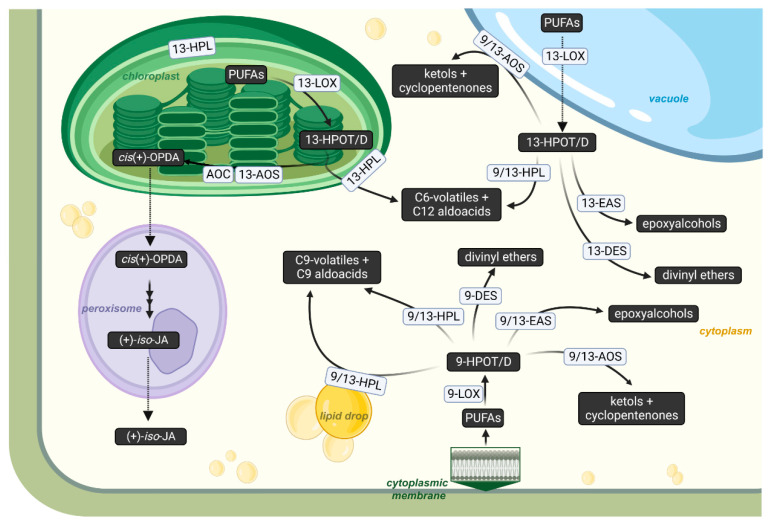
Scheme of intracellular compartmentalization of 9- and 13-LOX pathways in plant cells based on combined data on the localization of 9- and 13-LOX pathways collected from various plant species (*A. thaliana*, *C. sativus*, *H. vulgare*, *M. truncatula*, *P. argentatum*, *P. inflata*, *S. lycopersicum*, *S. tuberosum*, *V. vinifera*, etc.) [160,161,162,163,164,165,166,167,168,169,170,171,172,173,174]. AOC—allene oxide cyclase; *cis*(+)-OPDA—*cis*-(+)-12-oxo-phytodienoic acid; (+)-7-*iso*-JA—(+)-7-*iso*-jasmonoyl-L-isoleucine. This figure was created with BioRender.com (accessed on 27 November 2023).

Additionally, the haem-binding domain of CYP74s, compared to monooxygenases P450, contains an additional motif of nine amino acid residues (Figure 8) [159]. From an evolutionary point of view, the deletion of nine amino acid residues is a more likely event than analogous insertion. The hypothesis regarding the ancient origin of CYP74s is also supported by the fact that the localization of a large number of modern enzymes of the CYP74 family, namely CYP74A and CYP74B enzymes, is associated with chloroplasts (Figure 9), which are the descendants of the first photosynthetic organisms [160,161].

**Figure 10:**
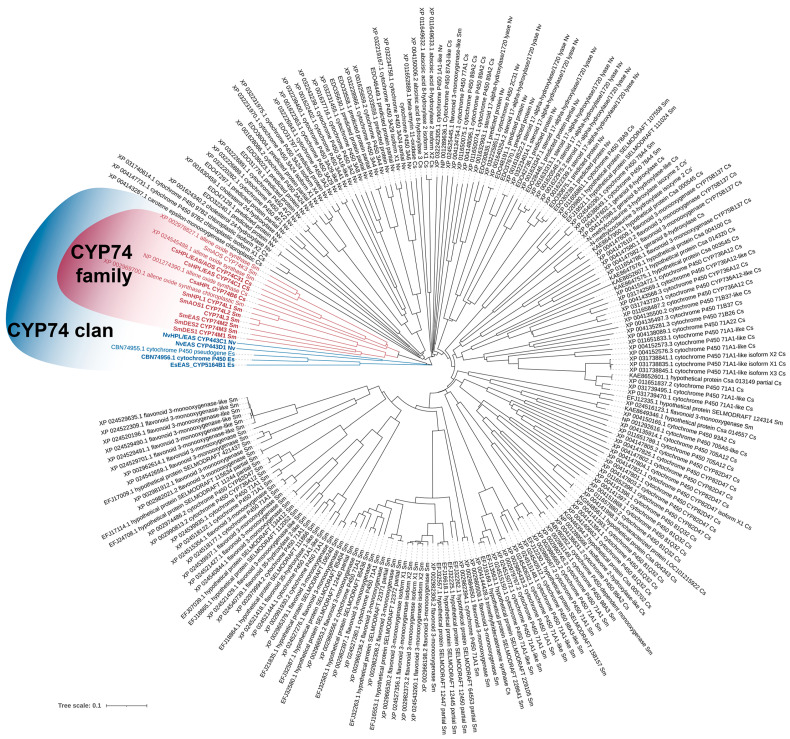
Phylogenetic tree of the P450s of *C. sativus* (Cs), *S. moellendorffii* (Sm), *N. vectensis* (Nv) and *E. siliculosus* (Es). Enzymes of the CYP74 family are highlighted in red and enzymes of the CYP74 clan are highlighted in blue. Enzymes characterized in our studies are indicated in bold. The evolutionary history was inferred using the Neighbor-Joining method. Evolutionary analyses were conducted in MEGA7. The iTOL tool (https://itol.embl.de/) was used to visualize the phylogenetic model output.

We constructed a phylogenetic tree of all cytochromes P450 of four model organisms phylogenetically distant from each other, namely *C. sativus*, *S. moellendorffii*, *N. vectensis*, and *E. siliculosus*, to establish the place occupied by the CYP74 clan on it. On this tree, the CYP74 enzymes go to the root (Figure 10). Previously, similar phylogenetic studies of the P450 superfamily were carried out by Professor D. Nelson [175]. On this tree, the CYP74 clan is also localized at the root. Thus, the results of phylogenetic analyses also confirm that CYP74 enzymes are probably vestigial representatives or direct descendants of ancient oxygen-independent cytochromes P450, which existed before the separation of the main eukaryotic lineages.

## 5. Conclusions

The lipoxygenase cascade takes its essential place among other vital systems, being the source of such widely studied oxylipins as jasmonates, GLVs, and traumatin in plants, as well as prostaglandins, prostacyclins, and thromboxanes in animals. However, many widespread products of this biochemical pathway remain poorly studied; the pathways of their biosynthesis and further transformation, as well as their biological properties, are still unknown. Such a situation was observed in the case of many epoxyalcohols, EAS products, which have been found in a wide range of organisms belonging to different taxa. The enzymes responsible for their biosynthesis remained unknown until recently, despite a detailed study of the lipoxygenase cascade, at least in a number of objects. In organisms in which epoxyalcohols, products of EAS activity, were previously discovered, the enzymes responsible for their formation have now been identified and described. True EASs belonging to both the CYP74 family and the CYP74 clan have been characterized. A number of CYP74s previously characterized or annotated as AOSs, HPLs, or DESs have been shown to exhibit additional EAS activity. This fact explains the absence of EASs in plants in which epoxyalcohols, products of the EAS activity, have been identified. At the same time, the presence of two activities in one enzyme (usually HPL and EAS) allows the plant to use a wider range of compounds involved in protection from environmental changes: healing, signaling, communicative, and directly protective (antimicrobial and fungicide). Since the CYP74 enzymes are among the fastest enzymes in nature, the biosynthesis of the entire set of compounds occurs almost immediately after stress.

In general, a number of EASs in plants and animals were characterized, as well as a significantly larger number of enzymes with other types of catalytic activity, including AOSs, HPLs, and DESs, which also exhibit EAS activity. Structural features, catalytic mechanisms, and the results of phylogenetic studies have shown that plant EASs belong to the CYP74 family, while non-plant EASs belong to other families within the CYP74 clan. The following pattern was established: in CYP74C enzymes, previously described or annotated as HPLs, as a rule, EAS activity is one of the two main forms of activity, while in CYP74B and CYP74D enzymes, EAS activity is an additional minor activity. Experiments using labeled ^18^O allowed the deciphering of the EAS catalytic mechanism. It was shown that this mechanism proceeds through (1) homolysis of the hydroperoxy group, (2) rearrangement of the resulting oxy radical to form an epoxyallylic radical, and (3) recombination of the epoxyallylic radical with the hydroxyl radical, resulting in the formation of an epoxyalcohol. Thus, EASs are isomerases. The results of phylogenetic studies indicated that EASs and enzymes exhibiting EAS activity as an additional activity are not grouped together but are evenly distributed throughout the tree. The detailed structure of oxiranyl carbinols synthesized by plant and metazoan enzymes indicated that plant EASs, as well as EsEAS, mainly synthesize (*S*,*S*,*S*)-epoxyalcohol epimers (*trans*-epoxides), while BfEAS and NvEAS synthesize (*S*,*R*,*S*)-stereoisomers with a *cis*-epoxide.

The synthesis of oxiranyl carbinols, the main EAS products, involves the prototypical transformation of fatty acid hydroperoxides. This is confirmed by the fact that the synthesis of oxiranyl carbinols occurs non-enzymatically in the presence of acids [97,98], transition metals [99,100,101], hemoproteins [102], and upon heating [103], but at a much lower rate than EASs. The CYP74 clan EASs have a conversion rate comparable to other CYP74s, which are considered one of the fastest enzymes in nature. Thus, it can be assumed that EASs are prototypical enzymes of the metabolism of fatty acid hydroperoxides.

The first EAS to be discovered was the lancelet CYP440A1 (BfEAS) enzyme, a member of the CYP74 clan. The discovery of CYP74 EASs in plants and brown algae means that these enzymes are a fairly ancient group of enzymes that appeared before the separation of the main evolutional branches.

The fundamental knowledge about the mechanisms of transformations of fatty acid hydroperoxides catalyzed by hemoproteins makes a significant contribution to understanding the functioning of the lipoxygenase cascade, the products of which, oxylipins, play an important role in cellular signaling and the adaptation of organisms to biogenic and abiogenic stress factors. Novel oxylipins, including signaling compounds that may be responsible for the adaptation of organisms to different factors, were described. The results obtained may further contribute to the development of methods for the production of biologically active oxylipins involved in chemical and biological protection, as well as new-generation pharmaceuticals.

## References

[1] Brodhun F., Feussner I. (2011). Oxylipins in Fungi. FEBS J..

[2] Savchenko T.V., Zastrijnaja O.M., Klimov V.V. (2014). Oxylipins and Plant Abiotic Stress Resistance. Biochem. Mosc..

[3] De Domenico S., Taurino M., Gallo A., Poltronieri P., Pastor V., Flors V., Santino A. (2019). Oxylipin Dynamics in Medicago Truncatula in Response to Salt and Wounding Stresses. Physiol. Plant..

[4] Christie W.W., Harwood J.L. (2020). Oxidation of Polyunsaturated Fatty Acids to Produce Lipid Mediators. Essays Biochem..

[5] Muñoz P., Munné-Bosch S. (2020). Oxylipins in Plastidial Retrograde Signaling. Redox Biol..

[6] Shipelin V.A., Sidorova Y.S. (2020). Oxylipins—biologically active substances of food. Vopr. Pitan..

[7] Savchenko T., Degtyaryov E., Radzyukevich Y., Buryak V. (2022). Therapeutic Potential of Plant Oxylipins. Int. J. Mol. Sci..

[8] Gorina S., Ogorodnikova A., Mukhtarova L., Toporkova Y. (2022). Gene Expression Analysis of Potato (*Solanum Tuberosum* L.) Lipoxygenase Cascade and Oxylipin Signature under Abiotic Stress. Plants.

[9] Esser-von Bieren J. (2019). Eicosanoids in Tissue Repair. Immunol. Cell Biol..

[10] Wallace J.L. (2019). Eicosanoids in the gastrointestinal tract. Br. J. Pharmacol..

[11] Mitchell J.A., Kirkby N.S. (2019). Eicosanoids, Prostacyclin and Cyclooxygenase in the Cardiovascular System. Br. J. Pharmacol..

[12] Calder P.C. (2020). Eicosanoids. Essays Biochem..

[13] Hoxha M., Zappacosta B. (2020). CYP-derived eicosanoids: Implications for rheumatoid arthritis. Prostaglandins Other Lipid Mediat..

[14] Yasukawa K., Okuno T., Yokomizo T. (2020). Eicosanoids in Skin Wound Healing. Int. J. Mol. Sci..

[15] Alvarez M.L., Lorenzetti F. (2021). Role of eicosanoids in liver repair, regeneration and cancer. Biochem. Pharmacol..

[16] Radmark O. (2022). Formation of eicosanoids and other oxylipins in human macrophages. Biochem. Pharmacol..

[17] Mukhtarova L.S., Brühlmann F., Hamberg M., Khairutdinov B.I., Grechkin A.N. (2018). Plant Hydroperoxide-Cleaving Enzymes (CYP74 Family) Function as Hemiacetal Synthases: Structural Proof of Hemiacetals by NMR Spectroscopy. Biochim. Biophys. Acta (BBA)-Mol. Cell Biol. Lipids.

[18] Itoh A., Howe G.A. (2001). Molecular Cloning of a Divinyl Ether Synthase: Identification as a CYP74 cytochrome P-450 *. J. Biol. Chem..

[19] Stumpe M., Kandzia R., Göbel C., Rosahl S., Feussner I. (2001). A Pathogen-Inducible Divinyl Ether Synthase (CYP74D) from Elicitor-Treated Potato Suspension Cells. FEBS Lett..

[20] Fammartino A., Cardinale F., Göbel C., Mène-Saffrané L., Fournier J., Feussner I., Esquerré-Tugayé M.-T. (2007). Characterization of a Divinyl Ether Biosynthetic Pathway Specifically Associated with Pathogenesis in Tobacco. Plant Physiol..

[21] Gogolev Y.V., Gorina S.S., Gogoleva N.E., Toporkova Y.Y., Chechetkin I.R., Grechkin A.N. (2012). Green Leaf Divinyl Ether Synthase: Gene Detection, Molecular Cloning and Identification of a Unique CYP74B Subfamily Member. Biochim. Biophys. Acta (BBA)-Mol. Cell Biol. Lipids.

[22] Werck-Reichhart D., Feyereisen R. (2000). Cytochromes P450: A success story. Genome Biol..

[23] Shengying L., Chaulagain M.R., Knauff A.R., Podust L.M., Montgomery J., Sherman D.H. (2009). Selective oxidation of carbolide C–H bonds by an engineered macrolide P450 monooxygenase. Proc. Natl. Acad. Sci. USA.

[24] Ortiz de Montellano P.R., Nelson S.D. (2011). Rearrangement reactions catalyzed by cytochrome P450s. Arch. Biochem. Biophys..

[25] Poulos T.L. (2014). Heme enzyme structure and function. Chem. Rev..

[26] Lee D.-S., Nioche P., Hamberg M., Raman C.S. (2008). Structural Insights into the Evolutionary Paths of Oxylipin Biosynthetic Enzymes. Nature.

[27] Smith W.L., Urade Y., Jakobsson P.-J. (2011). Enzymes of the Cyclooxygenase Pathways of Prostanoid Biosynthesis. Chem. Rev..

[28] Guengerich F.P., Munro A.W. (2013). Unusual cytochrome P450 enzymes and reactions. J. Biol. Chem..

[29] Proteau P.J., Gerwick W.H. (1993). Divinyl Ethers and Hydroxy Fatty Acids from Three Species of Laminaria (Brown Algae). Lipids.

[30] Jiang Z.-D., Gerwick W.H. (1997). Novel Oxylipins from the Temperate Red Alga *Polyneura latissima*: Evidence for an Arachidonate 9(*S*)-Lipoxygenase. Lipids.

[31] Nelson D.R., Goldstone J.V., Stegeman J.J. (2013). The Cytochrome P450 Genesis Locus: The Origin and Evolution of Animal Cytochrome P450s. Philos. Trans. R. Soc. B Biol. Sci..

[32] Medina S., Gil-Izquierdo Á., Durand T., Ferreres F., Domínguez-Perles R. (2018). Structural/Functional Matches and Divergences of Phytoprostanes and Phytofurans with Bioactive Human Oxylipins. Antioxidants.

[33] Domínguez-Perles R., Abellán Á., Leon D., Ferreres F., Guy A., Oger C., Galano J., Durand T. (2018). Sorting out the Phytoprostane and Phytofuran Profile in Vegetable Oils. Food Res. Int..

[34] Domínguez-Perles R., Gil-Izquierdo A., Ferreres F., Medina S. (2019). Update on Oxidative Stress and Inflammation in Pregnant Women, Unborn Children (Nasciturus), and Newborns—Nutritional and Dietary Effects. Free. Radic. Biol. Med..

[35] Pinciroli M., Domínguez-Perles R., Garbi M., Abellán A., Oger C., Durand T., Galano J.M., Ferreres F., Gil-Izquierdo A. (2018). Impact of Salicylic Acid Content and Growing Environment on Phytoprostane and Phytofuran (Stress Biomarkers) in *Oryza Sativa* L.. J. Agric. Food Chem..

[36] Pinciroli M., Domínguez-Perles R., Abellán Á., Bultel-Poncé V., Durand T., Galano J.M., Ferreres F., Gil-Izquierdo Á. (2019). Statement of Foliar Fertilization Impact on Yield, Composition, and Oxidative Biomarkers in Rice. J. Agric. Food Chem..

[37] Lipan L., Collado-González J., Domínguez-Perles R., Corell M., Bultel-Poncé V., Galano J.-M., Durand T., Medina S., Gil-Izquierdo Á., Carbonell-Barrachina Á. (2020). Phytoprostanes and Phytofurans-Oxidative Stress and Bioactive Compounds-in Almonds Are Affected by Deficit Irrigation in Almond Trees. J. Agric. Food Chem..

[38] Collado-González J., Cano-Lamadrid M., Pérez-López D., Carbonell-Barrachina Á.A., Centeno A., Medina S., Griñán I., Guy A., Galano J.-M., Durand T. (2020). Effects of Deficit Irrigation, Rootstock, and Roasting on the Contents of Fatty Acids, Phytoprostanes, and Phytofurans in Pistachio Kernels. J. Agric. Food Chem..

[39] Ramos L.L., Jiménez-Aspee F., Theoduloz C., Burgos-Edwards A., Domínguez-Perles R., Oger C., Durand T., Gil-Izquierdo Á., Bustamante L., Mardones C. (2019). Phenolic, Oxylipin and Fatty Acid Profiles of the Chilean Hazelnut (*Gevuina avellana*): Antioxidant Activity and Inhibition of pro-Inflammatory and Metabolic Syndrome-Associated Enzymes. Food Chem..

[40] Medina S., Gil-Izquierdo Á., Abu-Reidah I.M., Durand T., Bultel-Poncé V., Galano J.-M., Domínguez-Perles R. (2020). Evaluation of Phoenix Dactylifera Edible Parts and Byproducts as Sources of Phytoprostanes and Phytofurans. J. Agric. Food Chem..

[41] Martínez Sánchez S., Domínguez-Perles R., Montoro-García S., Gabaldón J.A., Guy A., Durand T., Oger C., Ferreres F., Gil-Izquierdo A. (2020). Bioavailable Phytoprostanes and Phytofurans from *Gracilaria longissima* Have Anti-Inflammatory Effects in Endothelial Cells. Food Funct..

[42] Grechkin A. (1998). Recent Developments in Biochemistry of the Plant Lipoxygenase Pathway. Prog. Lipid Res..

[43] Ogorodnikova A.V., Mukhitova F.K., Grechkin A.N. (2015). Oxylipins in the Spikemoss *Selaginella Martensii*: Detection of Divinyl Ethers, 12-Oxophytodienoic Acid and Related Cyclopentenones. Phytochemistry.

[44] Wasternack C., Feussner I. (2018). The Oxylipin Pathways: Biochemistry and Function. Annu. Rev. Plant Biol..

[45] Deboever E., Deleu M., Mongrand S., Lins L., Fauconnier M.-L. (2020). Plant–Pathogen Interactions: Underestimated Roles of Phyto-Oxylipins. Trends Plant Sci..

[46] Tanaka M., Koeduka T., Matsui K. (2021). Green Leaf Volatile-Burst in *Selaginella moellendorffii*. Front. Plant Sci..

[47] Grechkin A.N., Ogorodnikova A.V., Egorova A.M., Mukhitova F.K., Ilyina T.M., Khairutdinov B.I. (2018). Allene Oxide Synthase Pathway in Cereal Roots: Detection of Novel Oxylipin Graminoxins. ChemistryOpen.

[48] Chechetkin I.R., Blufard A.S., Yarin A.Y., Fedina E.O., Khairutdinov B.I., Grechkin A.N. (2019). Detection and Identification of Complex Oxylipins in Meadow Buttercup (*Ranunculus acris*) Leaves. Phytochemistry.

[49] Yu X., Zhang W., Zhang Y., Zhang X., Lang D., Zhang X. (2019). The Roles of Methyl Jasmonate to Stress in Plants. Funct. Plant Biol..

[50] Ruan J., Zhou Y., Zhou M., Yan J., Khurshid M., Weng W., Cheng J., Zhang K. (2019). Jasmonic Acid Signaling Pathway in Plants. Int. J. Mol. Sci..

[51] Zhang G., Zhao F., Chen L., Pan Y., Sun L., Bao N., Zhang T., Cui C.-X., Qiu Z., Zhang Y. (2019). Jasmonate-Mediated Wound Signalling Promotes Plant Regeneration. Nat. Plants.

[52] Chen X., Wang D.-D., Fang X., Chen X.-Y., Mao Y.-B. (2019). Plant Specialized Metabolism Regulated by Jasmonate Signaling. Plant Cell Physiol..

[53] Zhai Q., Li C. (2019). The Plant Mediator Complex and Its Role in Jasmonate Signaling. J. Exp. Bot..

[54] Hellmann E., Helariutta Y. (2019). Plant Genetics: Advances in Regeneration Pathways. Curr. Biol..

[55] Ueda M., Kaji T., Kozaki W. (2020). Recent Advances in Plant Chemical Biology of Jasmonates. Int. J. Mol. Sci..

[56] Wu X., Ye J. (2020). Manipulation of Jasmonate Signaling by Plant Viruses and Their Insect Vectors. Viruses.

[57] Gomi K. (2020). Jasmonic Acid: An Essential Plant Hormone. Int. J. Mol. Sci..

[58] Griffiths G. (2020). Jasmonates: Biosynthesis, Perception and Signal Transduction. Essays Biochem..

[59] Jang G., Yoon Y., Choi Y.D. (2020). Crosstalk with Jasmonic Acid Integrates Multiple Responses in Plant Development. Int. J. Mol. Sci..

[60] Zhuo M., Sakuraba Y., Yanagisawa S. (2020). A Jasmonate-Activated MYC2-Dof2.1-MYC2 Transcriptional Loop Promotes Leaf Senescence in Arabidopsis. Plant Cell.

[61] Ali M.S., Baek K.-H. (2020). Jasmonic Acid Signaling Pathway in Response to Abiotic Stresses in Plants. Int. J. Mol. Sci..

[62] Yang Z., Huang Y., Yang J., Yao S., Zhao K., Wang D., Qin Q., Bian Z., Li Y., Lan Y. (2020). Jasmonate Signaling Enhances RNA Silencing and Antiviral Defense in Rice. Cell Host Microbe.

[63] Zhao L., Li X., Chen W., Xu Z., Chen M., Wang H., Yu D. (2022). The Emerging Role of Jasmonate in the Control of Flowering Time. J. Exp. Bot..

[64] Bonner J., English J. (1937). Purification of traumatin, a plant wound hormone. Science.

[65] Noordermeer M.A., Veldink G.A., Vliegenthart J.F.G. (2001). Fatty Acid Hydroperoxide Lyase: A Plant Cytochrome P450 Enzyme Involved in Wound Healing and Pest Resistance. ChemBioChem.

[66] Stumpe M., Bode J., Göbel C., Wichard T., Schaaf A., Frank W., Frank M., Reski R., Pohnert G., Feussner I. (2006). Biosynthesis of C9-Aldehydes in the Moss *Physcomitrella patens*. Biochim. Biophys. Acta (BBA)-Mol. Cell Biol. Lipids.

[67] Bouwmeester H., Schuurink R.C., Bleeker P.M., Schiestl F. (2019). The Role of Volatiles in Plant Communication. Plant J..

[68] Stolterfoht H., Rinnofner C., Winkler M., Pichler H. (2019). Recombinant Lipoxygenases and Hydroperoxide Lyases for the Synthesis of Green Leaf Volatiles. J. Agric. Food Chem..

[69] Hashem C., Hochrinner J., Bürgler M.B., Rinnofner C., Pichler H., Winkler M. (2022). From linoleic acid to hexanal and hexanol by whole cell catalysis with a lipoxygenase, hydroperoxide lyase and reductase cascade in *Komagataella phaffii*. Front. Mol. Biosci..

[70] Matsui K., Engelberth J. (2022). Green leaf volatiles—The forefront of plant responses against biotic attack. Plant Cell Physiol..

[71] Yactayo-Chang J.P., Hunter C.T., Alborn H.T., Christensen S.A., Block A.K. (2022). Production of the Green Leaf Volatile (*Z*)-3-Hexenal by a *Zea mays* Hydroperoxide Lyase. Plants.

[72] Yan B., Zheng H., Sang Y., Wang Y., Sun J., Li F., Wang J., Wang X. (2022). A Single Amino Acid Substitution in MIL1 Leads to Activation of Programmed Cell Death and Defense Responses in Rice. Int. J. Mol. Sci..

[73] Zhou Z.W., Wu Q.Y., Yang Y., Hu Q.C., Wu Z.J., Huang H.Q., Lin H.Z., Lai Z.X., Sun Y. (2022). The Dynamic Change in Fatty Acids during the Postharvest Process of Oolong Tea Production. Molecules.

[74] Jo H.E., Song K., Kim J.G., Lee C.H. (2022). Non-targeted metabolomic analysis for the comparative evaluation of volatile organic compounds in 20 globally representative cucumber lines. Front. Plant Sci..

[75] Yue R., Zhang Z., Shi Q., Duan X., Wen C., Shen B., Li X. (2022). Identification of the key genes contributing to the LOX-HPL volatile aldehyde biosynthesis pathway in jujube fruit. Int. J. Biol. Macromol..

[76] Kaur I., Korrapati N., Bonello J., Mukherjee A., Rishi V., Bendigiri C. (2022). Biosynthesis of natural aroma compounds using recombinant whole-cell tomato hydroperoxide lyase biocatalyst. J. Biosci..

[77] Aratani Y., Uemura T., Hagihara T., Matsui K., Toyota M. (2023). Green leaf volatile sensory calcium transduction in Arabidopsis. Nat. Commun..

[78] Coenen A., Ferrer M., Jaeger K.E., Schörken U. (2023). Synthesis of 12-aminododecenoic acid by coupling transaminase to oxylipin pathway enzymes. Appl. Microbiol. Biotechnol..

[79] Kato T., Yamaguchi Y., Abe N., Uyehara T., Namai T., Kodama M., Shiobara Y. (1985). Structure and Synthesis of Unsaturaded Trihydroxy C18 Fatty: Acids in Rice Plants Suffering from Rice Blast Disease. Tetrahedron Lett..

[80] Kato T., Yamaguchi Y., Hirukawa T., Hoshino N. (1991). Structural Elucidation of Naturally Occurring 9, 12, 13-Trihydroxy Fatty Acids by a Synthetic Study. Agric. Biol. Chem..

[81] Hamberg M. (1999). An Epoxy Alcohol Synthase Pathway in Higher Plants: Biosynthesis of Antifungal Trihydroxy Oxylipins in Leaves of Potato. Lipids.

[82] Hamberg M. (2002). Biosynthesis of New Divinyl Ether Oxylipins in Ranunculus Plants. Lipids.

[83] Hamberg M., Olsson U. (2011). Efficient and Specific Conversion of 9-Lipoxygenase Hydroperoxides in the Beetroot. Formation of Pinellic Acid. Lipids.

[84] Jin J., Boeglin W.E., Cha J.K., Brash A.R. (2012). 8R-Lipoxygenase-catalyzed synthesis of a prominent *cis*-epoxyalcohol from dihomo-*γ*-linolenic acid: A distinctive transformation compared with *S*-lipoxygenases. J. Lipid Res..

[85] Wennman A., Oliw E.H. (2013). Secretion of Two Novel Enzymes, Manganese 9*S*-Lipoxygenase and Epoxy Alcohol Synthase, by the Rice Pathogen *Magnaporthe salvinii*. J. Lipid Res..

[86] Aghofack-Nguemezi J., Schwab W. (2013). Spatiotemporal Changes in the Content and Metabolism of 9, 12, 13—Trihydorxy-10(*E*)-Octadecenoic Acid in Tomato (*Solanum lycopersicum* L. CV Balkonsar) Fruits. J. Sci. Technol..

[87] d’Ippolito G., Nuzzo G., Sardo A., Manzo E., Gallo C., Fontana A. (2018). Lipoxygenases and Lipoxygenase Products in Marine Diatoms. Methods Enzymol..

[88] An J.-U., Hong S.-H., Oh D.-K. (2018). Regiospecificity of a Novel Bacterial Lipoxygenase from Myxococcus Xanthus for Polyunsaturated Fatty Acids. Biochim. Et Biophys. Acta (BBA)-Mol. Cell Biol. Lipids.

[89] An J.-U., Lee I.-G., Ko Y.-J., Oh D.-K. (2019). Microbial Synthesis of Linoleate 9 *S* -Lipoxygenase Derived Plant C18 Oxylipins from C18 Polyunsaturated Fatty Acids. J. Agric. Food Chem..

[90] Oliw E.H. (2020). Linoleate Diol Synthase Related Enzymes of the Human Pathogens *Histoplasma capsulatum* and *Blastomyces dermatitidis*. Arch. Biochem. Biophys..

[91] Oliw E.H. (2021). Fatty Acid Dioxygenase-Cytochrome P450 Fusion Enzymes of Filamentous Fungal Pathogens. Fungal Genet. Biol..

[92] Edin M.L., Yamanashi H., Boeglin W.E., Graves J.P., DeGraff L.M., Lih F.B., Zeldin D.C., Brash A.R. (2021). Epoxide Hydrolase 3 (*Ephx3*) Gene Disruption Reduces Ceramide Linoleate Epoxide Hydrolysis and Impairs Skin Barrier Function. J. Biol. Chem..

[93] Blée E., Schuber F. (1990). Efficient Epoxidation of Unsaturated Fatty Acids by a Hydroperoxide-Dependent Oxygenase. J. Biol. Chem..

[94] Hamberg M., Hamberg G. (1996). Peroxygenase-catalyzed fatty acid epoxidation in cereal seeds (sequential oxidation of linoleic acid into 9 (*S*), 12 (*S*), 13 (*S*)-trihydroxy-10 (*E*)-octadecenoic acid). Plant Physiol..

[95] Blée E., Flenet M., Boachon B., Fauconnier M.-L. (2012). A Non-Canonical Caleosin from Arabidopsis Efficiently Epoxidizes Physiological Unsaturated Fatty Acids with Complete Stereoselectivity. FEBS J..

[96] Garscha U., Oliw E.H. (2009). Leucine/valine residues direct oxygenation of linoleic acid by (10*R*)-and (8*R*)-dioxygenases: Expression and site-directed mutagenesis of (10*R*)-dioxygenase with epoxyalcohol synthase activity. J. Biol. Chem..

[97] Gardner H.W., Weisleder D., Nelson E.C. (1984). Acid catalysis of a linoleic acid hydroperoxide: Formation of epoxides by an intramolecular cyclization of the hydroperoxide group. J. Org. Chem..

[98] Gardner H., Nelson E.C., Tjarks L.W., England R.E. (1984). Acid-Catalyzed Transformation of 13(S)-Hydroperoxylinoleic Acid into Epoxyhydroxyoctadecenoic and Trihydroxyoctadecenoic Acids. Chem. Phys. Lipids.

[99] Gardner H.W. (1975). Decomposition of linoleic acid hydroperoxides. Enzymic reactions compared with nonenzymic. J. Agric. Food Chem..

[100] Gardner H.W., Kleiman R. (1981). Degradation of Linoleic Acid Hydroperoxides by a Cysteine FeCl3 Catalyst as a Model for Similar Biochemical Reactions: II. Specificity in Formation of Fatty Acid Epoxides. Biochim. Et Biophys. Acta (BBA)-Lipids Lipid Metab..

[101] Dix T.A., Marnett L.J. (1985). Conversion of Linoleic Acid Hydroperoxide to Hydroxy, Keto, Epoxyhydroxy, and Trihydroxy Fatty Acids by Hematin. J. Biol. Chem..

[102] Gardner H.W. (1989). Oxygen Radical Chemistry of Polyunsaturated Fatty Acids. Free. Radic. Biol. Med..

[103] Hamberg M., Gotthammar B. (1973). A New Reaction of Unsaturated Fatty Acid Hydroperoxides: Formation of 11-Hydroxy-12,13-Epoxy-9-Octadecenoic Acid from 13-Hydroperoxy-9,11-Octadecadienoic Acid. Lipids.

[104] Chang M.S., Boeglin W.E., Guengerich F.P., Brash A.R. (1996). Cytochrome P450-Dependent Transformations of 15R- and 15S-Hydroperoxyeicosatetraenoic Acids: Stereoselective Formation of Epoxy Alcohol Products. Biochemistry.

[105] Song W.C., Baertschi S.W., Boeglin W.E., Harris T.M., Brash A.R. (1993). Formation of Epoxyalcohols by a Purified Allene Oxide Synthase. Implications for the Mechanism of Allene Oxide Synthesis. J. Biol. Chem..

[106] Hughes R.K., Yousafzai F.K., Ashton R., Chechetkin I.R., Fairhurst S.A., Hamberg M., Casey R. (2008). Evidence for Communality in the Primary Determinants of CYP74 Catalysis and of Structural Similarities between CYP74 and Classical Mammalian P450 Enzymes. Proteins.

[107] Hoffmann I., Oliw E.H. (2013). Discovery of a Linoleate 9*S*-Dioxygenase and an Allene Oxide Synthase in a Fusion Protein of Fusarium Oxysporum. J. Lipid Res..

[108] Hoffmann I., Jernerén F., Oliw E.H. (2013). Expression of Fusion Proteins of Aspergillus Terreus Reveals a Novel Allene Oxide Synthase. J. Biol. Chem..

[109] Graveland A. (1970). Enzymatic Oxidations of Linoleic Acid and Glycerol-1-Monolinoleate in Doughs and Flour-Water Suspensions. J. Am. Oil Chem. Soc..

[110] Panossian A.G., Avetissian G.M., Mnatsakanian V.A., Batrakov S.G., Vartanian S.A., Gabrielian E.S., Amroyan E.A. (1983). Unsaturated Polyhydroxy Acids Having Prostaglandin-Like Activity from Bryonia Alba II. Major Components. Planta Med..

[111] Gorshkov V.Y., Toporkova Y.Y., Tsers I.D., Smirnova E.O., Ogorodnikova A.V., Gogoleva N.E., Parfirova O.I., Petrova O.E., Gogolev Y.V. (2021). Differential Modulation of the Lipoxygenase Cascade during Typical and Latent *Pectobacterium Atrosepticum* Infections. Ann. Bot..

[112] Toporkova Y.Y., Gorina S.S., Bessolitsyna E.K., Smirnova E.O., Fatykhova V.S., Brühlmann F., Ilyina T.M., Mukhtarova L.S., Grechkin A.N. (2018). Double Function Hydroperoxide Lyases/Epoxyalcohol Synthases (CYP74C) of Higher Plants: Identification and Conversion into Allene Oxide Synthases by Site-Directed Mutagenesis. Biochim. Biophys. Acta (BBA)-Mol. Cell Biol. Lipids.

[113] Gorina S.S., Iljina T.M., Mukhtarova L.S., Toporkova Y.Y., Grechkin A.N. (2022). Detection of Unprecedented CYP74 Enzyme in Mammal: Hydroperoxide Lyase CYP74C44 of the Bat *Sturnira Hondurensis*. Int. J. Mol. Sci..

[114] Grechkin A.N., Mukhtarova L.S., Latypova L.R., Gogolev Y., Toporkova Y.Y., Hamberg M. (2008). Tomato CYP74C3 Is a Multifunctional Enzyme Not Only Synthesizing Allene Oxide but Also Catalyzing Its Hydrolysis and Cyclization. ChemBioChem.

[115] Grechkin A.N., Lantsova N.V., Mukhtarova L.S., Khairutdinov B.I., Gorina S.S., Iljina T.M., Toporkova Y.Y. (2023). Distinct Mechanistic Behaviour of Tomato CYP74C3 and Maize CYP74A19 Allene Oxide Synthases: Insights from Trapping Experiments and Allene Oxide Isolation. Int. J. Mol. Sci..

[116] Toporkova Y.Y., Smirnova E.O., Mukhtarova L.S., Gorina S.S., Grechkin A.N. (2020). Catalysis by Allene Oxide Synthases (CYP74A and CYP74C): Alterations by the Phe/Leu Mutation at the SRS-1 Region. Phytochemistry.

[117] Toporkova Y.Y., Askarova E.K., Gorina S.S., Ogorodnikova A.V., Mukhtarova L.S., Grechkin A.N. (2020). Epoxyalcohol Synthase Activity of the CYP74B Enzymes of Higher Plants. Biochim. Biophys. Acta (BBA)-Mol. Cell Biol. Lipids.

[118] Gorina S.S., Mukhitova F.K., Ilyina T.M., Toporkova Y.Y., Grechkin A.N. (2019). Detection of Unprecedented Allene Oxide Synthase Member of CYP74B Subfamily: CYP74B33 of Carrot (*Daucus carota*). Biochim. Biophys. Acta (BBA)-Mol. Cell Biol. Lipids.

[119] Toporkova Y.Y., Smirnova E.O., Iljina T.M., Mukhtarova L.S., Gorina S.S., Grechkin A.N. (2020). The CYP74B and CYP74D Divinyl Ether Synthases Possess a Side Hydroperoxide Lyase and Epoxyalcohol Synthase Activities That Are Enhanced by the Site-Directed Mutagenesis. Phytochemistry.

[120] Gorina S.S., Toporkova Y.Y., Mukhtarova L.S., Chechetkin I.R., Khairutdinov B.I., Gogolev Y.V., Grechkin A.N. (2014). Detection and Molecular Cloning of CYP74Q1 Gene: Identification of *Ranunculus acris* Leaf Divinyl Ether Synthase. Biochim. Biophys. Acta (BBA)-Mol. Cell Biol. Lipids.

[121] Gorina S.S., Toporkova Y.Y., Mukhtarova L.S., Smirnova E.O., Chechetkin I.R., Khairutdinov B.I., Gogolev Y.V., Grechkin A.N. (2016). Oxylipin Biosynthesis in Spikemoss *Selaginella moellendorffii*: Molecular Cloning and Identification of Divinyl Ether Synthases CYP74M1 and CYP74M3. Biochim. Biophys. Acta (BBA)-Mol. Cell Biol. Lipids.

[122] Gorina S.S., Mukhtarova L.S., Iljina T.M., Toporkova Y.Y., Grechkin A.N. (2022). Detection of divinyl ether synthase CYP74H2 biosynthesizing (11*Z*)-etheroleic and (1′*Z*)-colnelenic acids in asparagus (*Asparagus officinalis* L.). Phytochemistry.

[123] Grechkin A.N., Ilyasov A.V., Hamberg M. (1997). On the mechanism of biosynthesis of divinyl ether oxylipins by enzyme from garlic bulbs. Eur. J. Biochem..

[124] Toporkova Y.Y., Smirnova E.O., Gorina S.S., Mukhtarova L.S., Grechkin A.N. (2018). Detection of the First Higher Plant Epoxyalcohol Synthase: Molecular Cloning and Characterisation of the CYP74M2 Enzyme of Spikemoss *Selaginella moellendorffii*. Phytochemistry.

[125] Toporkova Y.Y., Fatykhova V.S., Gorina S.S., Mukhtarova L.S., Grechkin A.N. (2019). Epoxyalcohol Synthase RjEAS (CYP74A88) from the Japanese Buttercup (*Ranunculus japonicus*): Cloning and Characterization of Catalytic Properties. Biochemistry.

[126] Froehlich J.E., Itoh A., Howe G.A. (2001). Tomato allene oxide synthase and fatty acid hydroperoxide lyase, two cytochrome P450s involved in oxylipin metabolism, are targeted to different membranes of chloroplast envelope. Plant Physiol..

[127] Toporkova Y.Y., Fatykhova V.S., Gogolev Y.V., Khairutdinov B.I., Mukhtarova L.S., Grechkin A.N. (2017). Epoxyalcohol Synthase of *Ectocarpus Siliculosus*. First CYP74-Related Enzyme of Oxylipin Biosynthesis in Brown Algae. Biochim. Biophys. Acta Mol. Cell Biol. Lipids.

[128] Toporkova Y.Y., Gorina S.S., Mukhitova F.K., Hamberg M., Ilyina T.M., Mukhtarova L.S., Grechkin A.N. (2017). Identification of CYP443D1 (CYP74 Clan) of *Nematostella vectensis* as a First Cnidarian Epoxyalcohol Synthase and Insights into Its Catalytic Mechanism. Biochim. Biophys. Acta Mol. Cell Biol. Lipids.

[129] Gorina S.S., Toporkova Y.Y., Mukhtarova L.S., Grechkin A.N. (2019). The CYP443C1 (CYP74 Clan) Cytochrome of Sea Anemone *Nematostella Vectensis*—The First Metazoan Enzyme Possessing Hydroperoxide Lyase/Epoxyalcohol Synthase Activity. Dokl. Biochem. Biophys..

[130] Toporkova Y.Y., Smirnova E.O., Lantsova N.V., Mukhtarova L.S., Grechkin A.N. (2021). Detection of the First Epoxyalcohol Synthase/Allene Oxide Synthase (CYP74 Clan) in the Lancelet (*Branchiostoma belcheri*, Chordata). Int. J. Mol. Sci..

[131] Toporkova Y.Y., Smirnova E.O., Mukhtarova L.S., Grechkin A.N. (2022). Lipoxygenase Pathway in Brown Algae: The Biosynthesis of Novel Oxylipins “ectocarpins” by Hydroperoxide Bicyclase CYP5164A3 of *Ectocarpus Siliculosus*. Biochim. Biophys. Acta Mol. Cell Biol. Lipids.

[132] Grechkin A.N., Lantsova N.V., Khairutdinov B.I., Toporkova Y.Y. (2021). Hydroperoxide Bicyclase CYP50918A1 of *Plasmodiophora Brassicae* (Rhizaria, SAR): Detection of Novel Enzyme of Oxylipin Biosynthesis. Biochim. Biophys. Acta Mol. Cell Biol. Lipids.

[133] Gotoh O. (1992). Substrate Recognition Sites in Cytochrome P450 Family 2 (CYP2) Proteins Inferred from Comparative Analyses of Amino Acid and Coding Nucleotide Sequences. J. Biol. Chem..

[134] Gorina S.S., Smirnova E.O., Mukhtarova L.S., Toporkova Y.Y., Grechkin A.N. (2018). Conversion of Tomato Allene Oxide Synthase LeAOS3 (CYP74C3) into Epoxyalcohol Synthase by Site-Directed Mutagenesis. Dokl. Biochem. Biophys..

[135] Toporkova Y.Y., Gogolev Y.V., Mukhtarova L.S., Grechkin A.N. (2008). Determinants Governing the CYP74 Catalysis: Conversion of Allene Oxide Synthase into Hydroperoxide Lyase by Site-Directed Mutagenesis. FEBS Lett..

[136] Toporkova Y.Y., Ermilova V.S., Gorina S.S., Mukhtarova L.S., Osipova E.V., Gogolev Y.V., Grechkin A.N. (2013). Structure-Function Relationship in the CYP74 Family: Conversion of Divinyl Ether Synthases into Allene Oxide Synthases by Site-Directed Mutagenesis. FEBS Lett..

[137] Li Y., Wei K. (2020). Comparative Functional Genomics Analysis of Cytochrome P450 Gene Superfamily in Wheat and Maize. BMC Plant Biol..

[138] Pandian B.A., Sathishraj R., Djanaguiraman M., Prasad P.V.V., Jugulam M. (2020). Role of Cytochrome P450 Enzymes in Plant Stress Response. Antioxidants.

[139] Hansen C.C., Nelson D.R., Møller B.L., Werck-Reichhart D. (2021). Plant Cytochrome P450 Plasticity and Evolution. Mol. Plant.

[140] Xu L.-H., Du Y.-L. (2018). Rational and Semi-Rational Engineering of Cytochrome P450s for Biotechnological Applications. Synth. Syst. Biotechnol..

[141] Nelson D.R. (2018). Cytochrome P450 Diversity in the Tree of Life. Biochim. Biophys. Acta Proteins Proteom..

[142] Omura T., Gotoh O. (2017). Evolutionary Origin of Mitochondrial Cytochrome P450. J. Biochem..

[143] Lacchini E., Venegas-Molina J., Goossens A. (2023). Structural and Functional Diversity in Plant Specialized Metabolism Signals and Products: The Case of Oxylipins and Triterpenes. Curr. Opin. Plant Biol..

[144] Rustgi S., Springer A., Kang C., von Wettstein D., Reinbothe C., Reinbothe S., Pollmann S. (2019). Allene oxide synthase and hydroperoxide lyase, two non-canonical cytochrome P450s in *Arabidopsis thaliana* and their different roles in plant defense. Int. J. Mol. Sci..

[145] Carella P. (2020). Xylem-Mobile Oxylipins Are Critical Regulators of Induced Systemic Resistance in Maize. Plant Cell.

[146] Wang K.-D., Borrego E.J., Kenerley C.M., Kolomiets M.V. (2020). Oxylipins Other Than Jasmonic Acid Are Xylem-Resident Signals Regulating Systemic Resistance Induced by Trichoderma Virens in Maize. Plant Cell.

[147] Berg-Falloure K.M., Kolomiets M.V. (2023). Ketols Emerge as Potent Oxylipin Signals Regulating Diverse Physiological Processes in Plants. Plants.

[148] Ruocco N., Albarano L., Esposito R., Zupo V., Costantini M., Ianora A. (2020). Multiple Roles of Diatom-Derived Oxylipins within Marine Environments and Their Potential Biotechnological Applications. Mar. Drugs.

[149] Masui H., Kondo T., Kojima M. (1989). An Antifungal Compound, 9,12,13-Trihydroxy-(E)-10-Octadecenoic Acid, from *Colocasia antiquorum* Inoculated with *Ceratocystis fimbriata*. Phytochemistry.

[150] Weber H., Chételat A., Caldelari D., Farmer E.E. (1999). Divinyl ether fatty acid synthesis in late blight–diseased potato leaves. Plant Cell.

[151] Toporkova Y.Y., Bessolitsyna E.K., Smirnova E.O., Gorina S.S., Petrova O.E., Mukhtarova L.S., Grechkin A.N. (2018). Antimicrobial Activity of Geometric Isomers of Etherolenic Acid—The Products of Plant Lipoxygenase Cascade. Dokl. Biochem. Biophys..

[152] Oldham M.L., Brash A.R., Newcomer M.E. (2005). The Structure of Coral Allene Oxide Synthase Reveals a Catalase Adapted for Metabolism of a Fatty Acid Hydroperoxide. Proc. Natl. Acad. Sci. USA.

[153] Koljak R., Boutaud O., Shieh B.H., Samel N., Brash A.R. (1997). Identification of a naturally occurring peroxidase-lipoxygenase fusion protein. Science.

[154] Varvas K., Järving I., Koljak R., Valmsen K., Brash A.R., Samel N. (1999). Evidence of a Cyclooxygenase-Related Prostaglandin Synthesis in Coral: The allene oxide pathway is not involved in prostaglandin biosynthesis. J. Biol. Chem..

[155] Lõhelaid H., Teder T., Tõldsepp K., Ekins M., Samel N. (2014). Up-regulated expression of AOS-LOXa and increased eicosanoid synthesis in response to coral wounding. PLoS ONE.

[156] Gao B., Boeglin W.E., Zheng Y., Schneider C., Brash A.R. (2009). Evidence for an Ionic Intermediate in the Transformation of Fatty Acid Hydroperoxide by a Catalase-Related Allene Oxide Synthase from the *Cyanobacterium acaryochloris* Marina. J. Biol. Chem..

[157] Teder T., Lõhelaid H., Boeglin W.E., Calcutt W.M., Brash A.R., Samel N. (2015). A Catalase-Related Hemoprotein in Coral Is Specialized for Synthesis of Short-Chain Aldehydes: Discovery of p450-type hydroperoxide lyase activity in a catalase. J. Biol. Chem..

[158] Mashhadi Z., Newcomer M.E., Brash A.R. (2016). The Thr–His connection on the distal heme of catalase-related hemoproteins: A hallmark of reaction with fatty acid hydroperoxides. ChemBioChem.

[159] Brash A.R. (2009). Mechanistic Aspects of CYP74 Allene Oxide Synthases and Related Cytochrome P450 Enzymes. Phytochemistry.

[160] Feussner I., Wasternack C. (2002). The Lipoxygenase Pathway. Annu. Rev. Plant Biol..

[161] Hughes R.K., De Domenico S., Santino A. (2009). Plant Cytochrome CYP74 Family: Biochemical Features, Endocellular Localisation, Activation Mechanism in Plant Defence and Improvements for Industrial Applications. ChemBioChem.

[162] Vick B.A., Zimmerman D.C. (1987). Pathways of fatty acid hydroperoxide metabolism in spinach leaf chloroplasts. Plant Physiol..

[163] Droillard M.J., Rouet-Mayer M.A., Bureau J.M., Lauriere C. (1993). Membrane-Associated and Soluble Lipoxygenase Isoforms in Tomato pericarp (Characterization and Involvement in Membrane Alterations). Plant Physiol..

[164] Royo J., Vancanneyt G., Pérez A.G., Sanz C., Störmann K., Rosahl S., Sánchez-Serrano J.J. (1996). Characterization of three potato lipoxygenases with distinct enzymatic activities and different organ-specific and wound-regulated expression patterns. J. Biol. Chem..

[165] Ishiguro S., Kawai-Oda A., Ueda J., Nishida I., Okada K. (2001). The DEFECTIVE IN ANTHER DEHISCIENCE gene encodes a novel phospholipase A1 catalyzing the initial step of jasmonic acid biosynthesis, which synchronizes pollen maturation, anther dehiscence, and flower opening in Arabidopsis. Plant Cell.

[166] Stelmach B.A., Müller A., Hennig P., Gebhardt S., Schubert-Zsilavecz M., Weiler E.W. (2001). A novel class of oxylipins, sn1-O-′12-oxophytodienoyl)-sn2-O-(hexadecatrienoyl)-monogalactosyl diglycerides, from *Arabidopsis thaliana*. J. Biol. Chem..

[167] Porta H., Rocha-Sosa M. (2002). Plant lipoxygenases. Physiological and molecular features. Plant Physiol..

[168] Kimura H., Yokota K. (2004). Characterization of metabolic pathway of linoleic acid 9-hydroperoxide in cytosolic fraction of potato tubers and identification of reaction products. Appl. Biochem. Biotechnol..

[169] Xu Y., Ishida H., Reisen D., Hanson M.R. (2006). Upregulation of a tonoplast-localized cytochrome P450 during petal senescence in Petunia inflate. BMC Plant Biol..

[170] Wasternack C. (2007). Jasmonates: An Update on Biosynthesis, Signal Transduction and Action in Plant Stress Response, Growth and Development. Ann. Bot..

[171] De Domenico S., Tsesmetzis N., Di Sansebastiano G.P., Hughes R.K., Casey R., Santino A. (2007). Subcellular localisation of Medicago truncatula 9/13-hydroperoxide lyase reveals a new localisation pattern and activation mechanism for CYP74C enzymes. BMC Plant Biol..

[172] Acosta I.F., Farmer E.E. (2010). Jasmonates. Arab. Book.

[173] Wasternack C., Song S. (2017). Jasmonates: Biosynthesis, metabolism, and signaling by proteins activating and repressing transcription. J. Exp. Bot..

[174] Li M., Yu G., Cao C., Liu P. (2021). Metabolism, signaling, and transport of jasmonates. Plant Commun..

[175] Nelson D., Werck-Reichhart D. (2011). A P450-Centric View of Plant Evolution. Plant J..

